# Asbestos Detection with Fluorescence Microscopy Images and Deep Learning

**DOI:** 10.3390/s21134582

**Published:** 2021-07-04

**Authors:** Changjie Cai, Tomoki Nishimura, Jooyeon Hwang, Xiao-Ming Hu, Akio Kuroda

**Affiliations:** 1 Department of Occupational and Environmental Health, University of Oklahoma Health Sciences Center, University of Oklahoma, Oklahoma City, OK 73069, USA; jooyeon-hwang@ouhsc.edu; 2 Unit of Biotechnology, Graduate School of Integrated Sciences for Life, Hiroshima University, Higashi-Hiroshima 739-8530, Japan; tn-nishimurat@hiroshima-u.ac.jp; 3 Center for Analysis and Prediction of Storms, University of Oklahoma, Norman, OK 73072, USA; xhu@ou.edu

**Keywords:** asbestos, fluorescence microscopy, Convolutional Neural Networks (CNN), YOLOv4

## Abstract

Fluorescent probes can be used to detect various types of asbestos (serpentine and amphibole groups); however, the fiber counting using our previously developed software was not accurate for samples with low fiber concentration. Machine learning-based techniques (e.g., deep learning) for image analysis, particularly Convolutional Neural Networks (CNN), have been widely applied to many areas. The objectives of this study were to (1) create a database of a wide-range asbestos concentration (0–50 fibers/liter) fluorescence microscopy (FM) images in the laboratory; and (2) determine the applicability of the state-of-the-art object detection CNN model, YOLOv4, to accurately detect asbestos. We captured the fluorescence microscopy images containing asbestos and labeled the individual asbestos in the images. We trained the YOLOv4 model with the labeled images using one GTX 1660 Ti Graphics Processing Unit (GPU). Our results demonstrated the exceptional capacity of the YOLOv4 model to learn the fluorescent asbestos morphologies. The mean average precision at a threshold of 0.5 (mAP@0.5) was 96.1% ± 0.4%, using the National Institute for Occupational Safety and Health (NIOSH) fiber counting Method 7400 as a reference method. Compared to our previous counting software (Intec/HU), the YOLOv4 achieved higher *accuracy* (0.997 vs. 0.979), particularly much higher *precision* (0.898 vs. 0.418), *recall* (0.898 vs. 0.780) and *F-1 score* (0.898 vs. 0.544). In addition, the YOLOv4 performed much better for low fiber concentration samples (<15 fibers/liter) compared to Intec/HU. Therefore, the FM method coupled with YOLOv4 is remarkable in detecting asbestos fibers and differentiating them from other non-asbestos particles.

## 1. Introduction

Asbestos is a fibrous silicate mineral that has been widely used in construction materials due to its useful properties, such as high ultimate tensile strength, low thermal conduction, and relative resistance to chemical attacks [1,2]. Since asbestos is composed of microscopic bundles of silicate fibers, asbestos fibers can become airborne when asbestos-containing materials (ACMs) are damaged mechanically or deteriorated by long-term exposure to sunlight. The inhalation of airborne asbestos fibers damages the lungs, resulting in serious health problems, such as pleural mesothelioma and lung cancers [3,4,5]. Asbestos-related diseases cause an estimated 255,000 deaths annually throughout the world [6], and the occurrence of asbestos-linked cancers contributes to rise [7,8]. Although the use of asbestos is now prohibited in many developed countries (e.g., U.S.), ACMs still remain in old buildings, thus producing airborne asbestos fibers. In the U.S., asbestos has been responsible for over 200,000 deaths in the last few decades [9]; even today, there are approximately 2000 to 3000 new cases of mesothelioma, an asbestos-related cancer, diagnosed every year [10]. U.S. has asbestos-related productivity losses of approximately 0.36% of annual GDP, or 86,100 million dollars losses caused by asbestos [6]. Therefore, taking measures against asbestos to prevent exposure is critical. 

The most commonly used method for air samples relies on phase-contrast microscopy (PCM). After making the filters transparent, optical PCM is used to count the fibers that are longer than 5 µm, thinner than 3 µm, and have aspect ratios larger than 3:1. However, PCM cannot easily distinguish asbestos fibers from other natural or man-made fibers with similar dimensions and has low sensitivity for thin chrysotile fibers [11,12,13]. PCM cannot distinguish between asbestos and non-asbestos fibers, which causes great uncertainty regarding the actual asbestos fiber concentration. Thus, PCM fiber counts may include a large fraction of non-asbestos fibers (referred to as contamination), and estimating the actual extent of contamination requires the differential counting of asbestos fibers. In Japan, any PCM fiber counts above 1 fiber per liter (f/L) triggers re-testing using either scanning electron microscopy (SEM) or transmission electron microscopy (TEM) [14]. In the U.S., TEM analysis is recommended if serious contamination from non-asbestos fibers occurs in samples (NIOSH Method 7402) [11,12,13]. Compared to PCM, TEM has greater resolution and thus can detect smaller fibers [1,15,16]; however, its application is high cost and time-consuming for sample preparation and analysis [17]. Polarized light microscopy (PLM) is another option that can differentiate asbestos from non-asbestos fiber, but its sensitivity is even lower than PCM [18], and thus it is only used for bulk samples (NIOSH 9000) [11,12,13]. 

Recently, we developed asbestos-specific fluorescent probes based on asbestos-binding proteins [19,20]. Fluorescent probes have sufficient affinity and specificity for detecting all asbestos types (serpentine and amphibole groups), and they can be used to distinguish asbestos from ten kinds of commonly used non-asbestos fibrous materials, except for silicon carbide whiskers [20]. The fluorescent labeling of asbestos trapped on a membrane filter is completed within ten minutes using such probes. Then, the fluorescent-labeled asbestos fibers are immediately visualized using fluorescent microscopy (FM) [19,20]. Furthermore, we evaluated the specificity of the FM method using practical samples collected from demolition sites. Although the FM method is not a NIOSH-approved method, it correctly identified approximately 95% of the fluorescent stained fibers, and it can differentiate asbestos from non-asbestos fibers (340 fluorescent fibers from 34 different samples) [21]. Thus, the FM method can become a practical asbestos monitoring technique in many occupational settings, as it does not require the electron microscopic identification of asbestos. However, automated fiber counting would still be required. 

Previously, we developed an asbestos counting software with algorithms for counting crossed and splayed fibers according to counting rules [18]. The developed software afforded automated counts, which showed a good correlation (r = 0.99) with the manual counts of the practical samples with medium to high fiber concentrations. However, the counts were much less accurate with a correction of r = 0.64 at low fiber concentrations (<15 fibers/liter), possibly because of the interfering autofluorescent dust particles [18]. In this study, to improve the fiber identification accuracy and enable quality control, we used a deep-learning artificially intelligent model that both professional and non-professional asbestos analysts can train using training data. 

The Convolutional Neural Networks (CNN) was used successfully in many areas, such as self-driving cars, disease diagnosis, and object detection and warning [22,23,24]. The goal of CNN is to reduce complex data arrays into simpler forms that are easier to process while retaining critical features. First, the image is scanned by a set of filters to reduce the image into smaller array sets (or a “feature map”, which captures the main unique object features in the image) within the convolutional layer. Then, a set of feature maps generated by the filters make up a convolutional layer. After the convolutional layer is generated, a pooling layer often follows in order to decrease the amount of computational power needed to analyze the data. The number of convolutional and pooling layers can vary depending on the structure of the network [25]. The final layer of the CNN is the fully connected layer, which predicts class possibilities. Most of the state-of-the-art CNN-based algorithms, such as Darknet, AlexNet, Resnet, and GoogleNet are open source codes [26,27], which can dramatically reduce the cost of applying them for development. 

One popular state-of-the-art CNN-based model for detecting objects in an image is “You Only Look Once version 4” or YOLOv4, which significantly improved from its previous versions in terms of both speed and accuracy [28]. YOLOv4 utilizes a new backbone, Cross Stage Partial Darknet53 (also known as CSPDarknet53), to enhance the learning capability of CNN [29]. In addition, YOLOv4 can be trained and used on conventional Graphics Processing Units (GPU) with 8–16 GB-VRAM, which can make its broad use possible. 

In this study, we prepared the asbestos samples in the laboratory to create the fluorescent image datasets for training and testing the deep learning model. We applied YOLOv4 to detect asbestos in a wide range of asbestos concentration (0–50 fibers/liter) sample images and then evaluated the model performances. We trained the model using a commercially available Graphics Processing Units (GPU): GeForce GTX 1660 Ti. Finally, we analyzed the potential methods of improving the prediction results. 

## 2. Materials and Methods

### 2.1. Sample Preparation

To prepare FM images of airborne asbestos fibers, airborne dust was filtered through a nitrocellulose membrane filter at various demolition sites in Japan. For the collection of relatively pure asbestos fibers, air was collected from an air-tight chamber in which crushed asbestos minerals were dispersed. Most of the concentrations of airborne asbestos at demolition sites were not high. Therefore, to fill up a gap, we added air dust samples generated using relatively pure asbestos minerals. A total of 60 air filter samples (40 samples were prepared from airborne dust at various demolition sites, and the others were from relatively pure asbestos minerals) were used in this study. The filter samples were stained using Asbester Air 2 kit by Siliconbio Inc. (Hiroshima, Japan). FM images were acquired using a BX60 microscope equipped with a DP70 camera (Olympus Corporation., Tokyo, Japan). The asbestos fibers (more than 5 µm in length and less than 3 µm in diameter) were marked based on a gold standard—the NIOSH fiber counting Method 7400 [11,12,13]. The counting of non-asbestos particles was assisted by imageJ software [30]. 

Finally, a total of 176 FM images (111 images containing asbestos fibers and non-asbestos fluorescent particles and 65 images containing only fluorescent particles) were obtained from 13 airborne dust samples and used for the training of YOLOv4. After learning, a total of 47 images (30 images containing asbestos fibers and non-asbestos fluorescent particles and the others containing only fluorescent particles) obtained from seven other airborne dust samples were used for testing of the trained YOLOv4 and Intec/HU asbestos counting software [18]. 

### 2.2. Flowchart and YOLOV4 Network Architecture to Detect Asbestos

**Figure 1:**
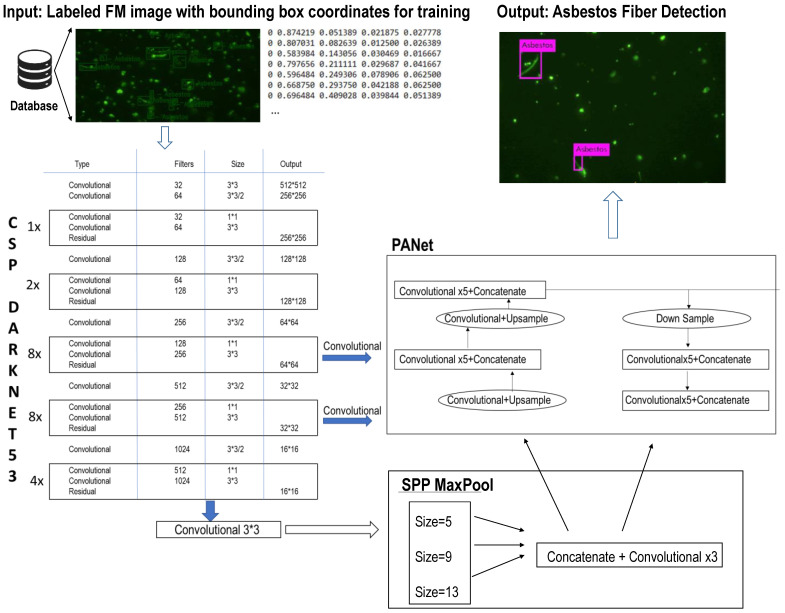
Flowchart and network architecture of YOLOv4 to detect asbestos.

The YOLOV4 outperforms the existing YOLOV3 for object detection in terms of having better accuracy and speed, which is achieved by using “Bag of Freebies” and “Bag of Specials” techniques [28]. The flowchart and network architecture of YOLOv4 to detect asbestos are shown in Figure 1.

We first annotated the FM images. The annotation process was completed by utilizing Yolo_mark, which is an open-source Graphical User Interface (GUI) that allows for objects to be marked within the images. The program was compiled in Microsoft Visual Studio (MSVS) 2015 (Microsoft Corporation, Albuquerque, NM, USA) to run on Windows Operating System (OS) with OpenCV. A text file was created with a list of the names and locations of each section image (see the Input section in Figure 1). In the text file, the first number identifies the object class, which is zero (0 = asbestos). The next two columns represent the X- and Y-coordinates of the object, and the last two columns determine the height and width of the bounding box. Then, the annotated images were used for training the YOLOv4 model to detect the asbestos fibers. The model was trained three times. 

YOLOv4 architecture can be broken down into three blocks assuming the “image” is passed as an input (see Figure 1). The first block (Backbone Network) is referred to “Feature Extraction” architecture. YOLOV4 implements the CSP backbone method, which has 53 convolutional layers for accurate image classification, also known as CSPDarknet53 [28]. CSP makes the separation of the bottom layer feature map into two different portions allowable with only one going through the dense block, and then the two recombine at the end and move to the next stage. Therefore, the CSP decreases the architecture complexity that allows a more efficient computation [29]. The second block (Neck) is referred to as “Feature Aggregation” architecture, which acts as an extra layer between the CSPDarknet53 backbone and head, so that it helps to blend and merge the features formed in the backbone block instead of following the traditional approach of CNN where everything is of linear form. YOLOv4 uses the Path Aggregation Network (PANet) for feature aggregation [31,32] and Spatial Pyramid Pooling (SPP) method to set apart the important features obtained from the backbone block. YOLOv4 Head is the third block, which uses dense prediction for anchor-based detection that helps in dividing the image into multiple cells and inspect each cell to find the probability of having an object using the post-processing techniques [33].

## 3. Results

### 3.1. Detection of Asbestos Fibers Using Deep Learning

The mean average precision (mAP) was 96.1% ± 0.4% at a threshold (or probability of detection) of 0.5 (@0.5). Table 1 summarizes the confusion matrix of the testing results. *Precision* represents the model’s ability to identify relevant data points that were classified as true and that were actually true. *Recall* is described as the model’s ability to find all relevant data points. It is the proportion of total correctly identified data points overall relevant data points. Maximizing *precision* often comes at the expense of *recall* and vice versa. Therefore, *F1-score* is considered the balance between *precision* and *recall*. Determining the *F1-score* is useful in this assessment to ensure optimal *precision* and *recall* scores can be achieved. Their calculations are as follows:

(1) $$Precision=\frac{TP}{TP+FP}$$

(2) $$Recall=\frac{TP}{TP+FN}$$

(3) $$F1=\frac{2*Recall*Precision}{Recall+Precision}$$

**Figure 2:**
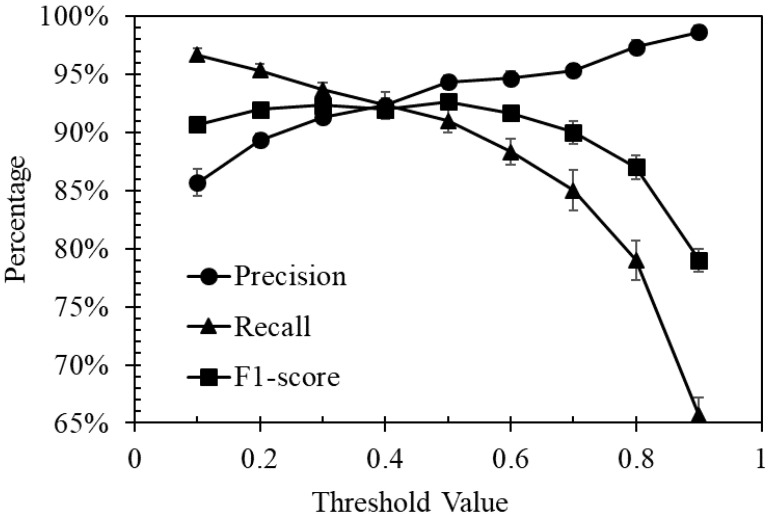
Evaluation metric change over threshold values.

The *precision*, *recall* and *F1-score* were 0.943 ± 0.006, 0.910 ± 0.010, 0.927 ± 0.006 at a threshold of 0.5, respectively. Figure 2 shows how the evaluation metric changes with various threshold values. Increasing the threshold from 0.1 to 0.9 caused the *precision* to increase from 0.857 ± 0.012 to 0.987 ± 0.006, while the *recall* score decreased from 0.967 ± 0.006 to 0.657 ± 0.015. *F1-score* reached the highest values of 0.927 ± 0.006 at a threshold value of 0.5. 

**Figure 3:**
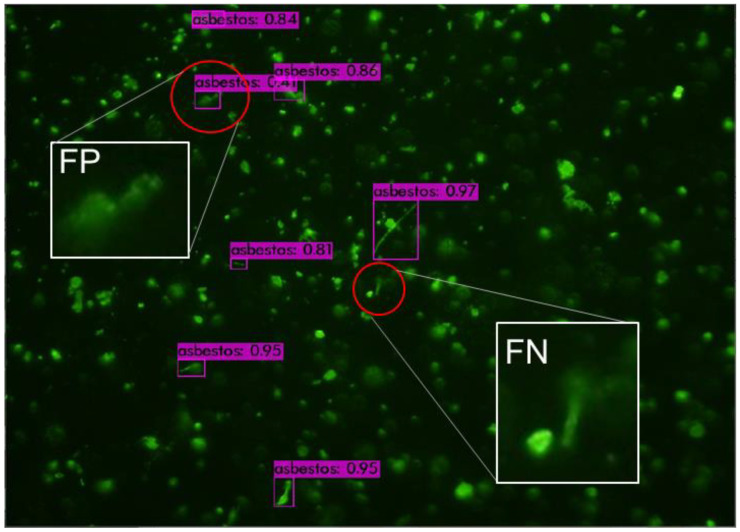
False positive (FP) and false negative (FN) examples.

**Figure 4:**
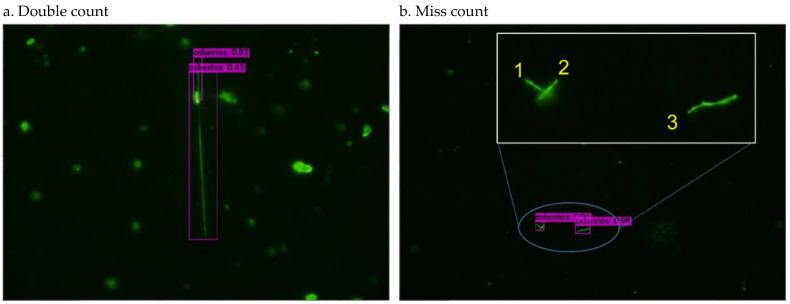
Double count (**a**) and miss count (**b**) examples.

Figure 3 shows an FM image, which includes both false positive and false negative cases. The model falsely recognized an aggregated particle as an asbestos fiber (FP case) and missed a fiber that has a blurring boundary together with other particles (FN case). Figure 4a double counts a single fiber due to the overlap with a non-fiber particle, and Figure 4b counted two vertically-crossed fibers (1 and 2) as one fiber. More images for training are needed to improve the model’s capability of recognizing asbestos in uncommon situations, as shown in Figure 3 and Figure 4.

### 3.2. Comparison of YOLOv4 to Intec/HU Using NIOSH 7400 as the Reference Method

We compared the YOLOv4 performance at the threshold value of 0.5 to our previously developed asbestos counting software—Intec/HU [18]—in Table 2. Most of the manually identified asbestos fibers were counted as “asbestos” in both Intec/HU asbestos counting software and the trained YOLOv4, while 13 asbestos fibers were miss-counted in the Intec/HU asbestos counting software, and 6 asbestos fibers were miss-counted in the trained YOLOv4 (Table 2), indicating that the false-negative rate was improved in the trained YOLOv4 model. The false positive rate was markedly decreased (6 from 64) in the trained YOLOv4 performance. The YOLOv4 performances were also tested at threshold values of 0.1 and 0.7 (Table 2). As expected, the *precision* scores slightly increased, and *recall* scores decreased as threshold values increased. Both *accuracy* and *F1-score* were almost the same at threshold values of 0.5 and 0.7. Overall, the YOLOv4 was found to be improved regarding *precision*, *recall*, and *F1-score* in comparison with Intec/HU asbestos counting software. 

**Figure 5:**
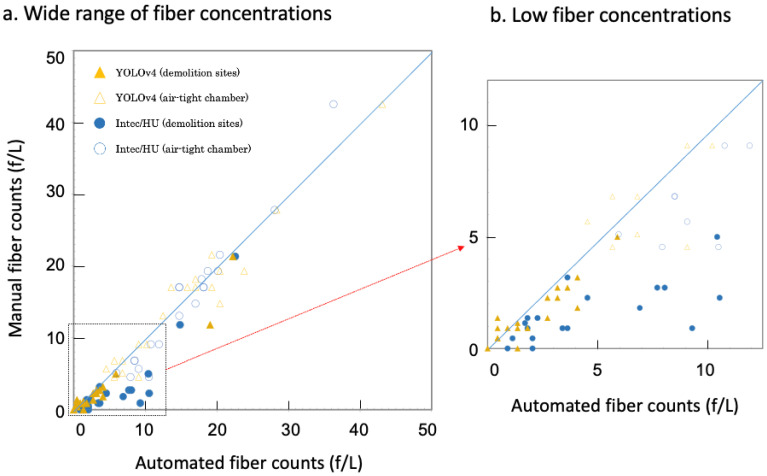
Correlation between manual and automated fiber counts at (**a**) the wide range of fiber concentrations (0–50 fibers/liter, f/L) and (**b**) low fiber concentrations (<15 f/L). The dotted area in (**a**) was enlarged in (**b**). Shaded and open triangles (brown) represent data points analyzed by YOLOv4 at threshold value of 0.7 with dust samples at demolition sites and relatively pure asbestos minerals, respectively. Shaded and open circles (blue) represent data points analyzed by Intec/HU asbestos software with dust samples at demolition sites and relatively pure asbestos minerals, respectively.

Next, we used 40 air filter samples (20 samples were prepared from airborne dust at various demolition sites and the others were from relatively pure asbestos minerals) in order to count asbestos fiber concentrations using the trained YOLOv4 and Intec/HU asbestos software. For the calculation of asbestos fiber concentrations, the total numbers of asbestos fibers counted using 100 FM images of each air filter sample were divided by collected air volumes. As we already reported [18], the counts of Intec/HU asbestos software showed less correlation (*r* = 0.768, Figure 5b) with the manual counts at low fiber concentration, although they performed a good correlation (*r* = 0.964) at the wide range of fiber concentrations (Figure 5a). In contrast, YOLOv4 showed much better performance at low fiber concentration (Figure 5b). Initially, we compared the counts of YOLOv4 with the manual counts at a threshold of 0.5 and obtained correlation (*r* = 0.925) at the wide range of fiber concentrations using dust samples at demolition sites. When we changed the threshold value of 0.7, the correlation factor increased to 0.968. Then, we included samples of relatively pure asbestos minerals and measured correlation at low fiber concentration (*r* = 0.921) as well as at the wide range of fiber concentrations (*r* = 0.979; Figure 5a,b, respectively). 

## 4. Discussion

In this work, we present a deep learning-based machine learning model that is able to provide end-to-end automation of the sample analysis process, starting with transforming the input FM images to recognizing and counting the number of asbestos fibers in the image. The main shortcoming of our previously developed asbestos counting software (Intec/HU) was the relatively high percentage of non-fibrous particles misidentified as fibers (low *precision*) that makes it not sufficient for its use in fully automated counting mode. Compared to the Intec/HU, the trained YOLOv4 at a threshold of 0.5 achieved higher *accuracy* (0.997 vs. 0.979), particularly much higher *precision* (0.898 vs. 0.418), *recall* (0.898 vs. 0.780), and *F-1 score* (0.898 vs. 0.544). In addition, the YOLOv4 performed much better for low fiber concentration samples compared to Intec/HU. 

Although our study demonstrates that the state-of-the-art deep learning model, YOLOv4 is a powerful tool to assist humans in recognizing asbestos fibers in FM images, additional work is still needed. For instance, the functions of calculating the fiber dimensions (such as length, width, aspect ratio, etc.) should be added in the future due to their significance in evaluating the health impacts. The semantic segmentation model will be able to solve these issues. For example, Frei and Kruis successfully used the mask region-based CNNs (or R-CNNs), which is the most widely used R-CNN for segmentation tasks, to automatically analyze the fiber shape [34]. In addition, PCM is the most commonly used method recommended by NIOSH method 7400 (or 7402); however, PCM does not provide an easy way to differentiate asbestos and non-asbestos fibers. If the air contains significant amounts of non-asbestos fibers, FM-based testing will provide more reliable estimates of asbestos contamination, which results in implications for risk assessment and epidemiological studies based on this method. Therefore, the NIOSH method 7400 (or 7402) could be improved in the future. 

## Figures and Tables

**Table 1:** Confusion matrix of the testing results.

Threshold = 0.5		Actual Value	
		True	False
YOLOv4 Predicted	True	TP = 428 ± 5	FP = 27 ± 3
	False	FN = 43 ± 8	TN = 0 ± 0

**Table 2:** Confusion matrix of the testing results.

		Intec/HU		YOLOv4					
				Threshold = 0.1		Threshold = 0.5		Threshold = 0.7	
		Asbestos	Non-Asbestos	Asbestos	Non-Asbestos	Asbestos	Non-Asbestos	Asbestos	Non-Asbestos
Manually counted (NIOSH 7400)	Asbestos	46	13	54	5	53	6	52	7
	Non-asbestos	64	3586	12	3638	6	3644	5	3645
*Accuracy*		0.979		0.995		0.997		0.997	
*Precision*		0.418		0.818		0.898		0.912	
*Recall*		0.780		0.915		0.898		0.881	
*F1-score*		0.544		0.864		0.898		0.897	

## Data Availability

All data, results, and source codes in this study are free to download from the Github website at: https://github.com/OEH-AI-CAI/Asbestos_Detection (accessed on 4 July 2021).

## References

[1] Mossman B.T., Bignon J., Corn M., Seaton A., Gee J.B. (1990). Asbestos: Scientific developments and implications for public policy. Science.

[2] Perkins R.L., Harvey B.W. (1993). Method for the Determination of Asbestos in Bulk Building Materials.

[3] Davis J.M., Beckett S.T., Bolton R.E., Collings P., Middleton A.P. (1978). Mass and number of fibres in the pathogenesis of asbestos-related lung disease in rats. Br. J. Cancer.

[4] Ramos-Nino M.E., Testa J.R., Altomare D.A., Pass H.I., Carbone M., Bocchetta M., Mossman B.T. (2006). Cellular and molecular parameters of mesothelioma. J. Cell. Biochem..

[5] Kanarek M.S. (2011). Mesothelioma from chrysotile asbestos: Update. Ann. Epidemiol..

[6] Furuya S., Chimed-Ochir O., Takahashi K., David A., Takala J. (2018). Global asbestos disaster. Int. J. Environ. Res. Public Health.

[7] Theakston F. (2000). Asbestos. Air quality guidelines for Europe.

[8] Furuya S., Takahashi K. (2017). Experience of Japan in achieving a total ban on asbestos. Int. J. Environ. Res. Public Health.

[9] Landrigan P.J., Lemen R.A. (2018). Asbestos related diseases in the United States: Historical trends and current situation. Int. Collab..

[10] Pease D.F., Kratzke R.A. (2017). Oncolytic viral therapy for mesothelioma. Front. Oncol..

[11] NIOSH. Health. Division of Physical Sciences (1994). NIOSH, Manual of Analytical Methods (No. 94-113).

[12] NIOSH. Health. Division of Physical Sciences (2011). NIOSH, Manual of Analytical Methods (No. 94-113).

[13] NIOSH. Health. Division of Physical Sciences (2019). NIOSH, Manual of Analytical Methods (No. 94-113).

[14] JES Center (2012). Solid Waste Management and Recycling Technology of Japan.

[15] Kauffer E., Billon-Galland M.A., Vigneron J.C., Veissiere S., Brochard P. (1996). Effect of preparation methods on the assessment of airborne concentrations of asbestos fibres by transmission electron microscopy. Ann. Occup. Hyg..

[16] Perry A. (2004). A Discussion of Asbestos Detection Techniques for Air and Soil.

[17] Taylor D.G., Baron P.A., Shulman S.A., Carter J.W. (1984). Identification and counting of asbestos fibers. Am. Ind. Hyg. Assoc. J..

[18] Alexandrov M., Ichida E., Nishimura T., Aoki K., Ishida T., Hirota R., Ikeda T., Kawasaki T., Kuroda A. (2015). Development of an automated asbestos counting software based on fluorescence microscopy. Environ. Monit. Assess..

[19] Kuroda A., Nishimura T., Ishida T., Hirota R., Nomura K. (2008). Detection of chrysotile asbestos by using a chrysotile-binding protein. Biotechnol. Bioeng..

[20] Ishida T., Alexandrov M., Nishimura T., Hirota R., Ikeda T., Kuroda A. (2013). Molecular engineering of a fluorescent bioprobe for sensitive and selective detection of amphibole asbestos. PLoS ONE.

[21] Nishimura T., Alexandrov M., Ishida T., Hirota R., Ikeda T., Sekiguchi K., Kuroda A. (2016). Differential Counting of Asbestos Using Phase Contrast and Fluorescence Microscopy. Ann. Occup. Hyg..

[22] Bojarski M., Del Testa D., Dworakowski D., Firner B., Flepp B., Goyal P., Jackel L.D., Monfort M., Muller U., Zhang J. (2016). End to end learning for self-driving cars. arXiv.

[23] Sun Y., Liu Y., Wang G., Zhang H. (2017). Deep learning for plant identification in natural environment. Comput. Intell. Neurosci..

[24] George J., Skaria S., Varun V.V. (2018). Using YOLO based deep learning network for real time detection and localization of lung nodules from low dose CT scans. Medical Imaging 2018: Computer-Aided Diagnosis.

[25] Huang G., Liu Z., Van Der Maaten L., Weinberger K.Q. Densely connected convolutional networks. Proceedings of the IEEE Conference on Computer Vision and Pattern Recognition.

[26] Redmon J., Divvala S., Girshick R., Farhadi A. You only look once: Unified, real-time object detection. Proceedings of the IEEE Conference on Computer Vision and Pattern Recognition.

[27] Iandola F.N., Han S., Moskewicz M.W., Ashraf K., Dally W.J., Keutzer K. (2016). SqueezeNet: AlexNet-level accuracy with 50× fewer parameters and <0.5 MB model size. arXiv.

[28] Bochkovskiy A., Wang C.Y., Liao H.Y.M. (2020). Yolov4: Optimal speed and accuracy of object detection. arXiv.

[29] Wang C.Y., Bochkovskiy A., Liao H.Y.M. (2020). Scaled-YOLOv4: Scaling Cross Stage Partial Network. arXiv.

[30] Schneider C.A., Rasband W.S., Eliceiri K.W. (2012). NIH Image to ImageJ: 25 years of image analysis. Nat. Methods.

[31] Liu S., Qi L., Qin H., Shi J., Jia J. Path aggregation network for instance segmentation. Proceedings of the IEEE Conference on Computer Vision and Pattern Recognition.

[32] Wang K., Liew J.H., Zou Y., Zhou D., Feng J. Panet: Few-shot image semantic segmentation with prototype alignment. Proceedings of the IEEE/CVF International Conference on Computer Vision.

[33] Redmon J., Farhadi A. (2018). Yolov3: An incremental improvement. arXiv.

[34] Frei M., Kruis F.E. (2021). FibeR-CNN: Expanding Mask R-CNN to improve image-based fiber analysis. Powder Technol..

